# Immunohistological Study of Monkey Foveal Retina

**DOI:** 10.1038/s41598-019-41793-y

**Published:** 2019-03-27

**Authors:** Tsunehiko Ikeda, Kimitoshi Nakamura, Hidehiro Oku, Taeko Horie, Teruyo Kida, Shinji Takai

**Affiliations:** 1 Department of Ophthalmology, Osaka Medical College, Takatsuki-City, Osaka Japan; 2 Nakamura Eye Clinic, Matsumoto-City, Nagano Japan; 3 Drug Discovery Medicine, Osaka Medical College, Takatsuki-City, Osaka Japan

## Abstract

The fovea centralis, an anatomically concave pit located at the center of the macula, is avascular, hypoxic, and characteristic of stem-cell niches of other tissues. We hypothesized that in the fovea, undifferentiated retinal-stem-cell-like cells may exist, and that neurogenesis may occur. Hence, we performed an immunohistological study using cynomolgus monkey retinas. After preparing frozen tissue sections of the retina including the foveal pit, immunostaining was performed for glial fibrillary acidic protein (GFAP), nestin, vimentin, neuron-specific class III β-tubulin (Tuj-1), arrestin 4, neurofilament, CD117, CD44, Ki67, and cellular retinaldehyde-binding protein (CRALBP), followed by fluorescence and/or confocal microscopy examinations. Immunostaining of the tissue sections enabled clear observation of strongly GFAP-positive cells that corresponded to the inner-half layer of the foveolar Müller cell cone. The surface layer of the foveal slope was partially costained with GFAP and vimentin. Tuj-1-positive cells were observed in the innermost layer of the foveolar retina, which spanned to the surrounding ganglion cell layer. Moreover, colocalization of Tuj-1 and GFAP was observed at the foveal pit. The coexpression of CD117 and CD44 was found in the interphotoreceptor matrix of the fovea. The foveolar cone stained positive for both nestin and arrestin 4, however, the photoreceptor layer outside of the foveola displayed weak staining for nestin. Colocalization of nestin and vimentin was observed in the inner half of the Henle layer, while colocalization of nestin and neurofilament was observed in the outer half, predominantly. Scattered Ki67-positive cells were observed in the cellular processes of the outer plexiform layer and the ganglion cell layer around the foveola. Immunostaining for CRALBP was negative in most parts of the GFAP-positive area. The Müller cell cone was divided into GFAP-strongly positive cells, presumably astrocytes, in the inner layer and nestin-positive/GFAP-weakly positive radial glia-like cells in the outer layer. These findings indicated that groups of such undifferentiated cells in the foveola might be involved in maintaining morphology and regeneration.

## Introduction

Reports in recent years have indicated the presence of stem cells in the central nervous system (CNS) and that neurogenesis is sustained into adulthood, thus attracting interest with respect to regenerative medicine^1–3^. Even in the sensory retina, which is part of the CNS, retinal stem cells capable of differentiating into neurons, glial cells, and photoreceptor cells are reportedly present in the so-called ‘ciliary marginal zone’ (CMZ) in both fish and amphibians, with regeneration occurring even into adulthood^4,5^. Although the adult mammalian retina had for long been considered to lack a neurodegenetive capacity, Martínez-Navarrete *et al*. recently revealed that gradual neurogenesis occurs in the peripheral retina of the primates throughout life^6^. In the CNS, the regions where the neurogenesis from the neural stem cells occurs, *i.e*. hippocampal subgranular zone and the subventricular zone/olfactory pathway^1–3^, undergo massive remodeling in neurodegenerative diseases, *e.g*. Alzheimer’s disease and Parkinson’s disease^7–9^. The foveola and its vicinity are the regions that most frequently involve in retinal neurodegenerative diseases, *e.g*. age-related macular degeneration, macular dystrophy, macular telangiectasia type 2^10–12^. It has been reported that neurodegenerative diseases are caused by dysfunction and loss of the neural stem cells^13,14^. Therefore, the reason why the fovea is the site of predilection of the neurodegenerative diseases might be that the retinal stem/progenitor cells reside in the foveal region, thus maintaining the tissue homeostasis by compensatory proliferation. Furthermore, the fovea is the only region where the closure of the retinal tissue defect takes place without scar formation, which is observed during repair of the macular hole^15^. It has been reported that scarless wound healing resembles to epimorphosis^16^ that is observed in the lens and retina regeneration of the adult newt^17^. In epimorphic regeneration, tissue resident stem/progenitor cells are recruited to the site of injury, then proliferate and differentiate to regain former morphology^18^. This evidence also supports our conjecture that retinal stem/progenitor cells reside in the foveal region.

In previous studies using tissue sections of monkey eyes, we observed that the outer layer of the foveola dominantly stained for nestin, a marker of neural stem cells, and that the level of nestin expression was higher in the macula than in the rest of the retina based on real-time polymerase chain reaction (PCR) results, thus suggesting a relationship of immature neural cells in the adult fovea to idiopathic macular hole closure via vitreous surgery^19,20^. In this present study, immunostaining of the foveal-region in monkey retinas was performed with markers for neural stem cells and differentiated glia and neurons to investigate the mechanism of neural differentiation in the retinal foveola and its vicinity.

## Results

### GFAP and nestin

**Figure 1:**
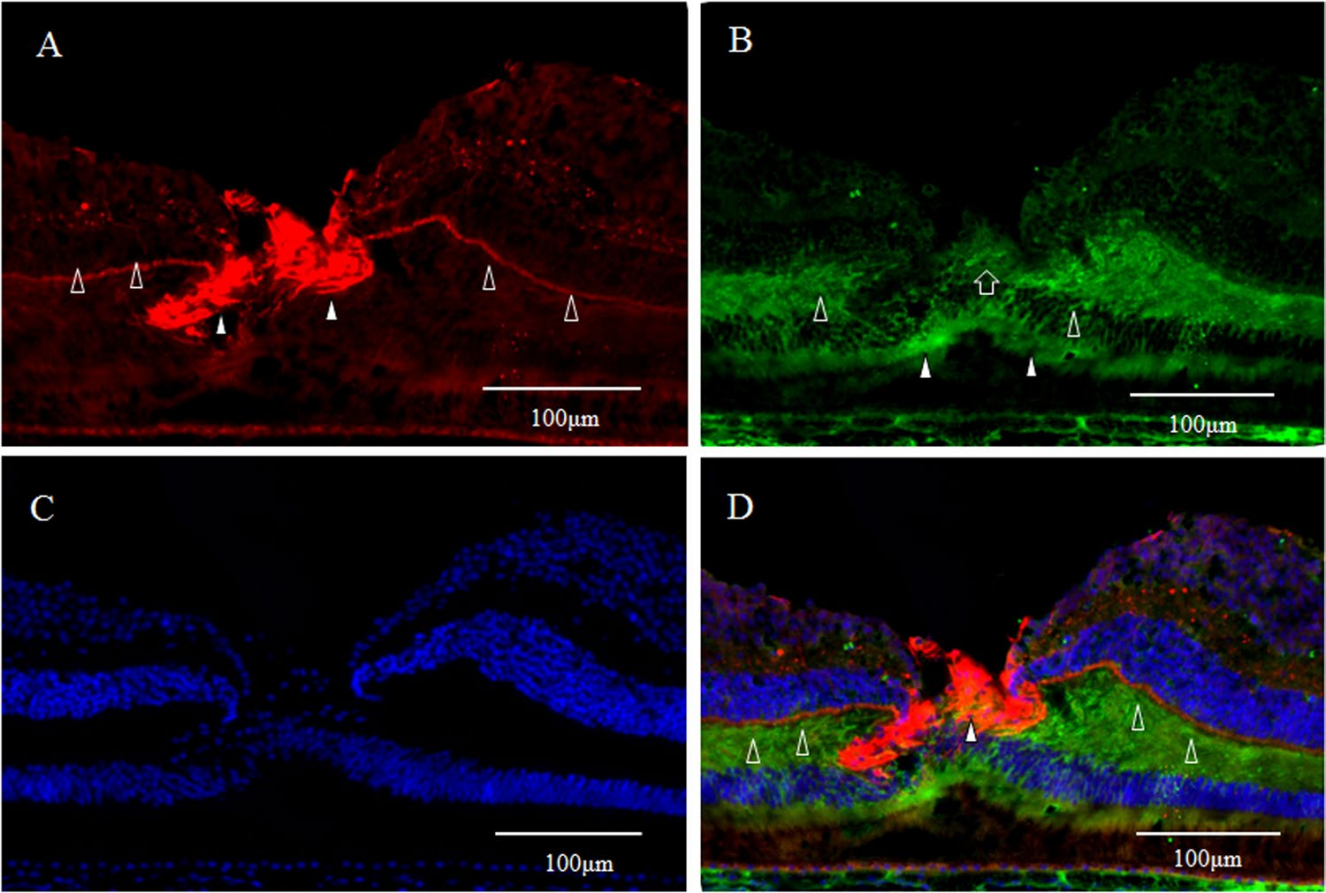
High-power-magnification optical microscopy images of a 6-year-old female monkey showing double-immunostaining of the fovea (vertical section) for glial fibrillary acidic protein (GFAP) and nestin. (**A**) Intense immunostaining for GFAP (red) expression is visible in the region where the inner-half of Müller cell cone is believed to be present (white arrowheads), and outer-half of Müller cell cone in the photoreceptor cell layer is less stained. At the boundary between the inner nuclear layer and the outer plexiform layer in the fovea, GFAP-positive cells span to join the GFAP-positive cells in the Müller cell cone (unfilled arrowheads). (**B**) Immunostaining for nestin (green) can be seen mainly in the region where the photoreceptor cell layer (white arrowheads) is located, with the center part of the Müller cell cone displaying weak staining (unfilled arrow). The nestin-positive region spans to the Henle layer (unfilled arrowheads). (**C**) Nuclear staining using 4′, 6-diamidino-2-phenylindole dihydrochloride (DAPI) (blue). (**D**) With double-immunostaining for GFAP and nestin, fluorescence microscopy shows a little overlapping (white arrowheads). At the boundary between the inner nuclear layer and the outer plexiform layer in the fovea, GFAP-positive cells span to join the GFAP-positive cells in the Müller cell cone (unfilled arrowheads).

GFAP expression (red) was detected in a vertical section of the fovea. However, the Müller cell cone was partially stained, with intense staining observed in the inner-half layer, excluding the photoreceptor cell layer (Fig. 1A, white arrowheads). Moreover, the GFAP-positive staining spanned to the area where the deep retinal capillary plexus at the border between the inner nuclear layer and the outer plexiform layer was believed to be present (Fig. 1A, unfilled arrowheads). Immunostaining for nestin (green) was observed mainly in the photoreceptor layer of the foveola (Fig. 1B, white arrowheads), and in the surrounding Henle layer (Fig. 1B, unfilled arrowheads), with weak staining observed in the inner layer of the Müller cell cone (Fig. 1B, unfilled arrow). A comparison with 4′, 6-diamidino-2-phenylindole dihydrochloride (DAPI) staining (Fig. 1C) showed that the nestin-positive region spanned to even the Henle layer. In the same section, fluorescence microscopy revealed weak double-staining for the GFAP and nestin regions at the center of the Müller cell cone (Fig. 1D, white arrowhead).

**Figure 2:**
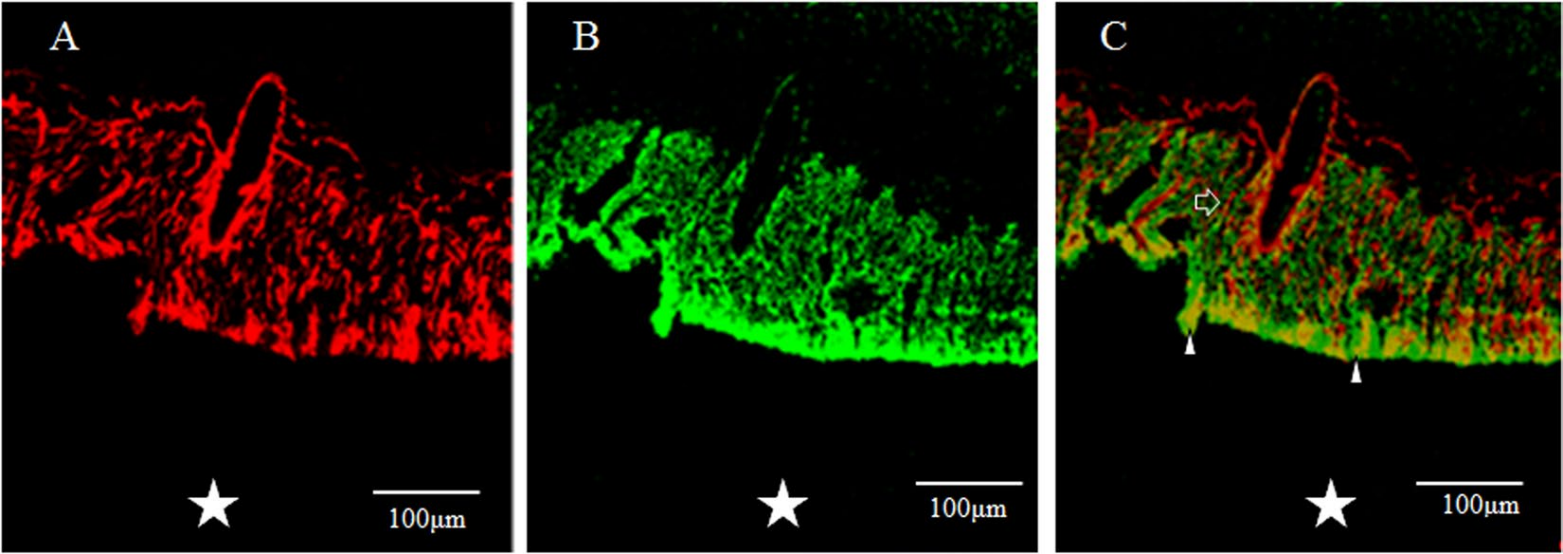
High-power-magnification optical microscopy images of a 2-year-old male monkey showing immunostaining of the foveal slope (horizontal cross section) for GFAP and nestin. (white stars demonstrate foveal pit). (**A**) A horizontal section of the inner layer of the foveal pit. Cells that stain positive for GFAP (red) surround the foveal pit in a radial manner. (**B**) A horizontal section of the inner layer of the foveal pit. Cells that stain positive for nestin (green) surround the foveal pit in a radial manner, (**C**) There are the regions that are slightly merged with GFAP and nestin (white arrowheads). Fibrous cells that express GFAP are also visible in the margin of the blood vessels around the foveola (unfilled arrow), which differs from the Müller cell morphologically.

**Figure 3:**
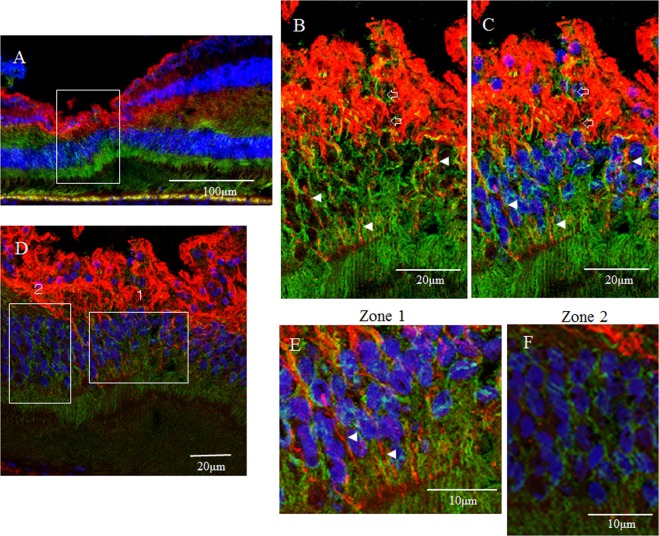
Confocal microscopy images of a 6-year-old female monkey showing double-immunostaining of the foveola (vertical cross section) for GFAP (red) and nestin (green), and nuclear staining with DAPI (blue). (**A**) Overview section of foveal pit. Square indicates B and C. (**B**,**C**) GFAP-positive elongated cells appear to run nearly vertical in the foveola, and some of them, while weakly stained, reach the photoreceptor cell layer (white arrowheads). In the inner layer of the foveola on the same section, the vertically aligned GFAP-positive cells appear to reach the shallow layer of the foveola and are partially merged with nestin. (unfilled arrows). (**D**) Magnified overview of foveal pit. Zone 1 indicates E and Zone 2 indicates F. (**E**) GFAP-positive elongated cells are visible in the foveolar (white arrowhead). (**F**) On the other hand, in the area around the foveola, these GFAP-positive cells are not observed.

In a horizontal section of the fovea, the GFAP-positive region (Fig. 2A) and the nestin-positive region (Fig. 2B) were partially merged (Fig. 2C, white arrowheads), showing GFAP-positive, outreaching, elongated projections clinging to blood vessels (Fig. 2C, unfilled arrow). Furthermore, under confocal microscopy (Fig. 3A), the GFAP-positive elongated cells appeared to run nearly vertical in the fovea, and some of those cells, although weakly stained, reached the photoreceptor cell layer (Fig. 3B,C, white arrowheads). In the inner layer of the fovea on the same section, the nearly vertical GFAP-positive elongated cells appeared to reach the shallow layer of the fovea (Fig. 3B,C, unfilled arrows). However, the GFAP-positive elongated cells were not observed in the area around the foveola (Fig. 3E,F).

**Figure 4:**
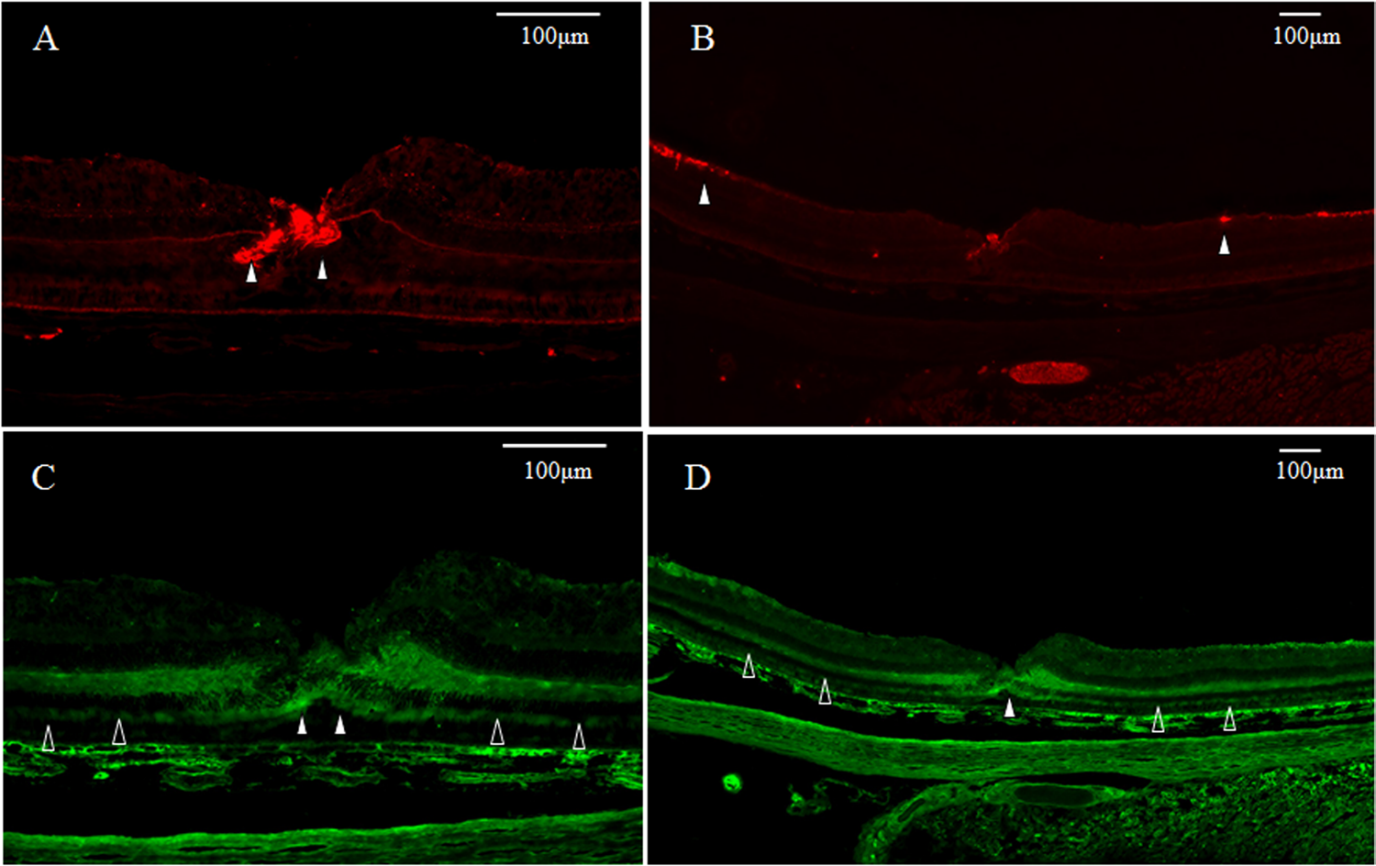
Medium- and low-power-magnification optical microscopy images of a 6-year-old female monkey showing immunostaining of the macula (vertical cross section) for GFAP and nestin. (**A**) GFAP-positive region (red) displays weak immunostaining, except the area where the inner layer of the Müller cell cone is located under medium-power magnification (white arrowheads). (**B**) The ganglion cell layer in the periphery shows partial staining for GFAP under low-power magnification (white arrowheads). (**C**,**D**) Under both medium- and low-power magnifications, the foveolar photoreceptor layer shows intense immunostaining for nestin (green) (white arrowheads), whereas the periphery shows weak immunostaining (unfilled arrowheads).

**Figure 5:**
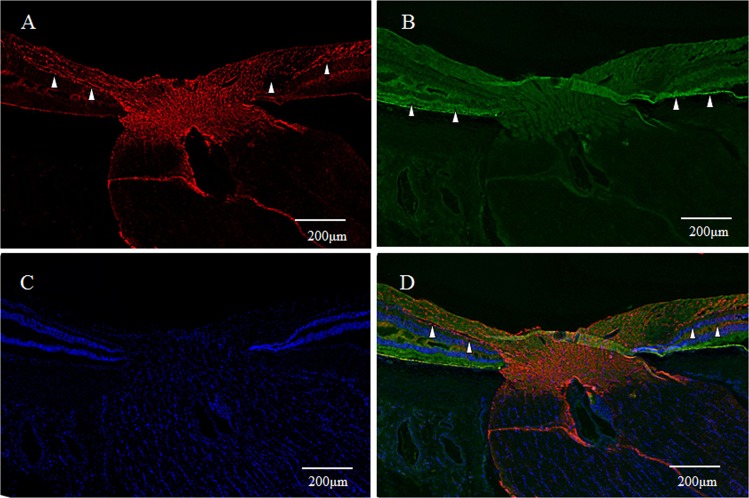
Medium-power-magnification optical microscopy images of a 2-year-old male monkey showing immunostaining of the optic disc (vertical cross section) for GFAP and nestin. (**A**) Image showing the region of the optic disc believed to be comprised of astrocytes and the retinal inner layer in the peripapillary area displaying immunostaining for GFAP (red) (white arrowheads). (**B**) The retinal outer layer is stained primarily for nestin (green) (white arrowheads). (**C**) DAPI staining (blue). (**D**) The immunostaining for GFAP is visible in the deep capillary plexus (white arrowheads). The optic disc periphery shows a similar staining pattern to that of the foveolar retina.

**Figure 6:**
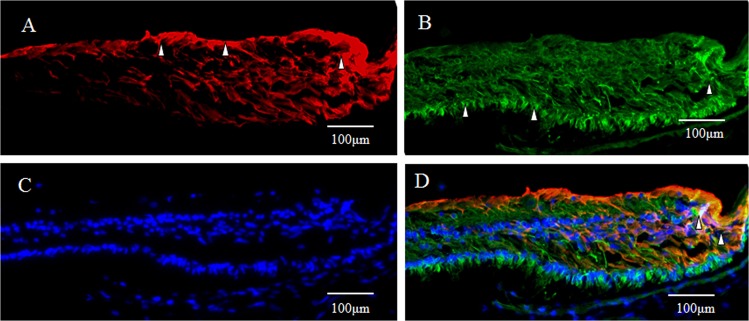
Medium-power-magnification optical microscopy images of a 2-year-old male monkey showing immunostaining of the retinal extreme periphery (so-called ciliary marginal zone) for GFAP and nestin. (**A**) In the retinal extreme periphery, the retinal inner layer expresses high levels of GFAP (red) (white arrowheads). (**B**) The retinal outer layer expresses high levels of nestin (green) (white arrowheads). (**C**) DAPI staining (blue). (**D**) In the same section, weak double-staining for GFAP and nestin region is visible near the ciliary body, showing a similar staining pattern as the foveolar retina (white arrowheads).

The GFAP-positive region outside of the internal layer of the Müller cell cone appeared weakly stained under medium-power magnification (Fig. 4A, white arrowheads), however, some intense staining was observed in the surrounding retinal inner layer under low-power magnification (Fig. 4B, white arrowheads). The nestin-positive region, under both medium- and low-power magnifications, appeared to be strongly stained in the outer layer of the foveola (Fig. 4C,D, white arrowheads), yet weakly stained in a region from the surrounding photoreceptor cell layer to the outer plexiform layer (Fig. 4C,D, unfilled arrowheads). In addition, in the area where astrocytes of the optic disc are believed to be present, as well as at the periphery of the optic disc, the immunostaining for GFAP was dominant in the retinal inner layer (Fig. 5A, white arrowheads) and around the deep capillary plexus (Fig. 5D, white arrowheads), whereas the immunostaining for nestin was dominant in the outer layer (Fig. 5B, white arrowheads) and showed the same staining property as observed in the foveal retina. The same findings were also observed in the CMZ of the extreme periphery of the retina (Fig. 6A,B, white arrowheads). In that same section, fluorescence microscopy revealed weak double-staining for the GFAP and nestin regions near the ciliary body (Fig. 6D, white arrowheads).

### GFAP and vimentin

**Figure 7:**
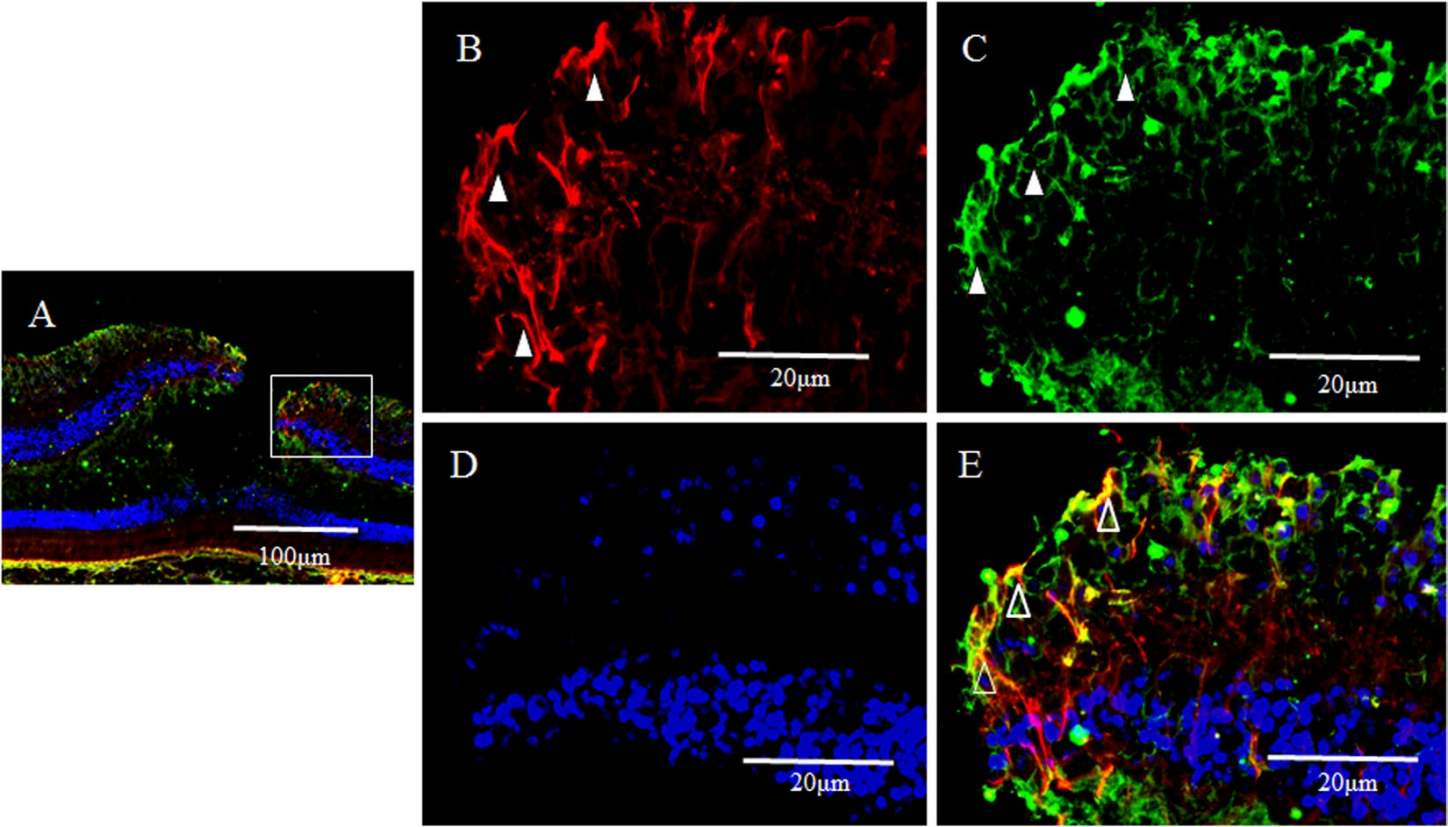
Medium and high-power-magnification optical microscopy images of a 2-year-old male monkey showing double-immunostaining of the fovea (vertical section) for GFAP and vimentin. (**A**) Medium-power-magnification optical microscopy images. (**B**) Immunostaining for GFAP (red) is observed in the inner layer of the fovea (white arrowheads). (**C**) Vimentin (green) is widely observed from the inner layer to outer layer in the fovea (white arrowheads). (**D**) DAPI staining (blue). (**E**) Weak double-staining for GFAP and vimentin is observed in the surface layer of the foveal slope (unfilled arrowheads).

As described above, intense immunostaining for GFAP (red) was observed in the inner-half layer of the Müller cell cone (Fig. 7B, white arrowheads). Immunostaining for vimentin (green) was widely observed from the inner to outer layer of the fovea (Fig. 7C, white arrowheads). Weak double-staining for GFAP and vimentin was observed at the foveal slope (Fig. 7E, white arrowheads). Immunostaining for GFAP and vimentin was not observed in the photoreceptor layer.

### GFAP and Tuj-1

**Figure 8:**
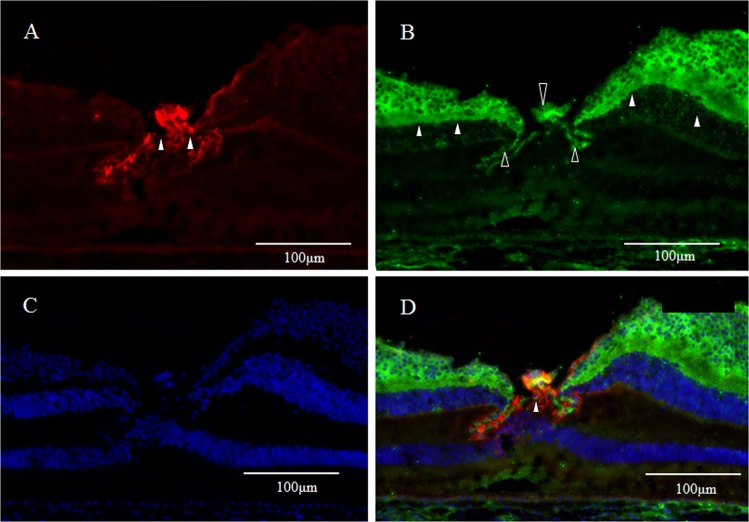
Medium-power-magnification optical microscopy images of a 4-year-old female monkey showing double-immunostaining of the foveola (vertical cross section) for GFAP and Tuj-1. (**A**) GFAP (red) expression is visible as intense staining in the inner layer of the foveolar Müller cell cone (white arrowheads), the same as is shown in Fig. 1. (**B**) Intense immunostaining for Tuj-1 (green) is visible in the inner retinal layer, particularly in the retinal ganglion cell layer (white arrowheads). However, definitive staining is also visible in the innermost layer of the foveal pit (unfilled arrowheads), which spans to the retinal ganglion cell layer. (**C**) DAPI staining (blue). (**D**) Yellowish regions, colocalization of GFAP and Tuj-1,are observed in the surface layer of the foveal pit. (white arrowhead).

**Figure 9:**
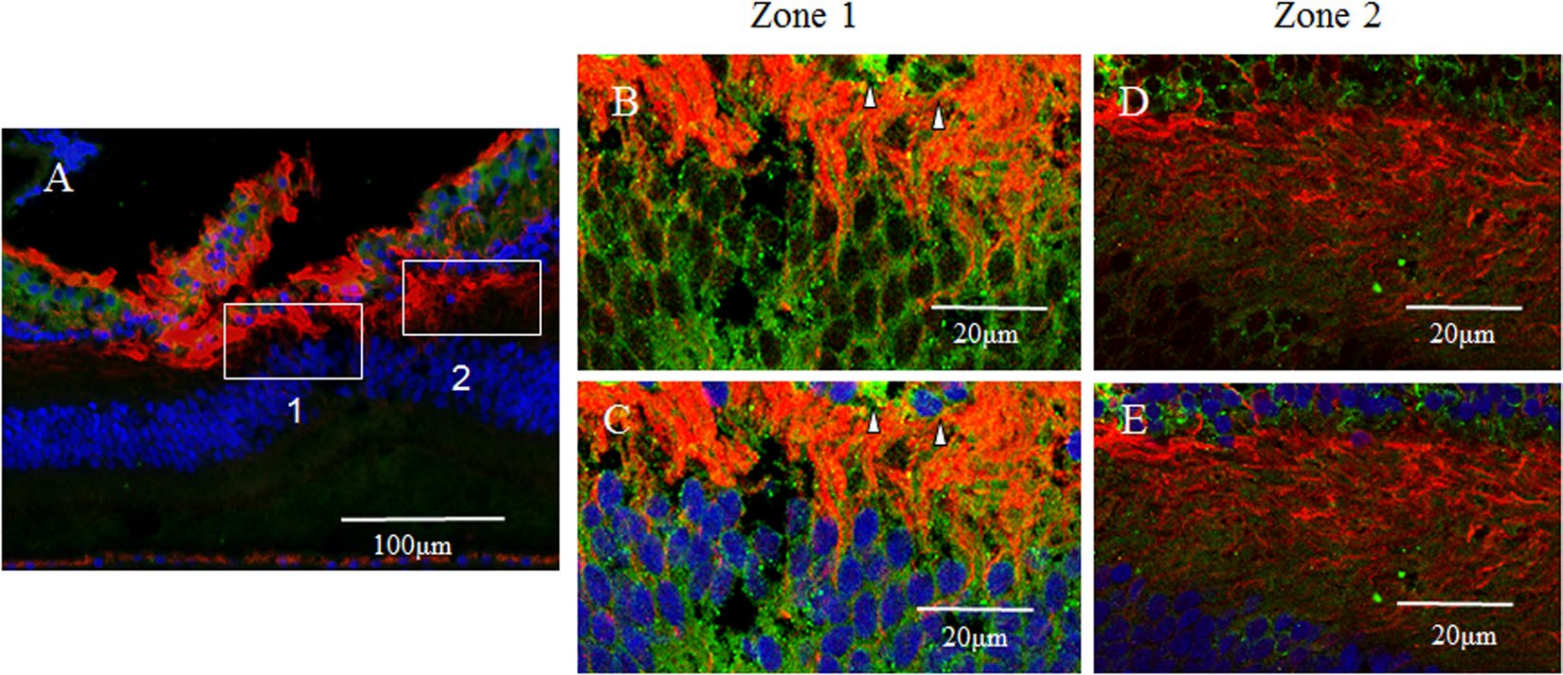
Confocal microscopy images of a 4-year-old female monkey showing immunostaining of the foveola (vertical cross section) for GFAP and Tuj-1 and nuclear staining for DAPI (blue). (**A**) Overview section of the foveal pit. Zone 1 indicates **B** and **C**. Zone 2 indicates **D** and **E**. (**B**,**C**) GFAP (red) is merged with Tuj-1 (green) in the innermost layer of the foveola (Zone 1, white arrowheads). (**D**,**E**) No clear evidence of such merging is visible in the area around the foveola (Zone 2, white arrowheads).

As described above, intense immunostaining for GFAP (red) was observed in the inner-half layer of the Müller cell cone in the foveola (Fig. 8A, white arrowheads). Immunostaining for Tuj-1 (green) was observed in the retinal inner layer, with intense staining particularly in the retinal ganglion cell layer (Fig. 8B, white arrowheads). However, definitive staining was also observed in the innermost layer of the foveal pit (Fig. 8B, unfilled arrowheads). In a section double-stained for a combination, including DAPI, the presence of Tuj-1-positive cells were observed in the innermost layer over the foveal pit area (Fig. 8D, white arrowhead), and multiple rows of ganglion cells were found to have migrated to that area (Fig. 8D). Moreover, a yellow-tone staining, believed to be the merging of Tuj-1 and GFAP, was observed at the foveal pit (Fig. 8D, white arrowhead). Confocal microscopy observation with magnification of the area of the fovea showed slight merging of GFAP and Tuj-1 in the innermost layer of the fovea (Fig. 9B,C, white arrowheads).

### Nestin and arrestin 4

**Figure 10:**
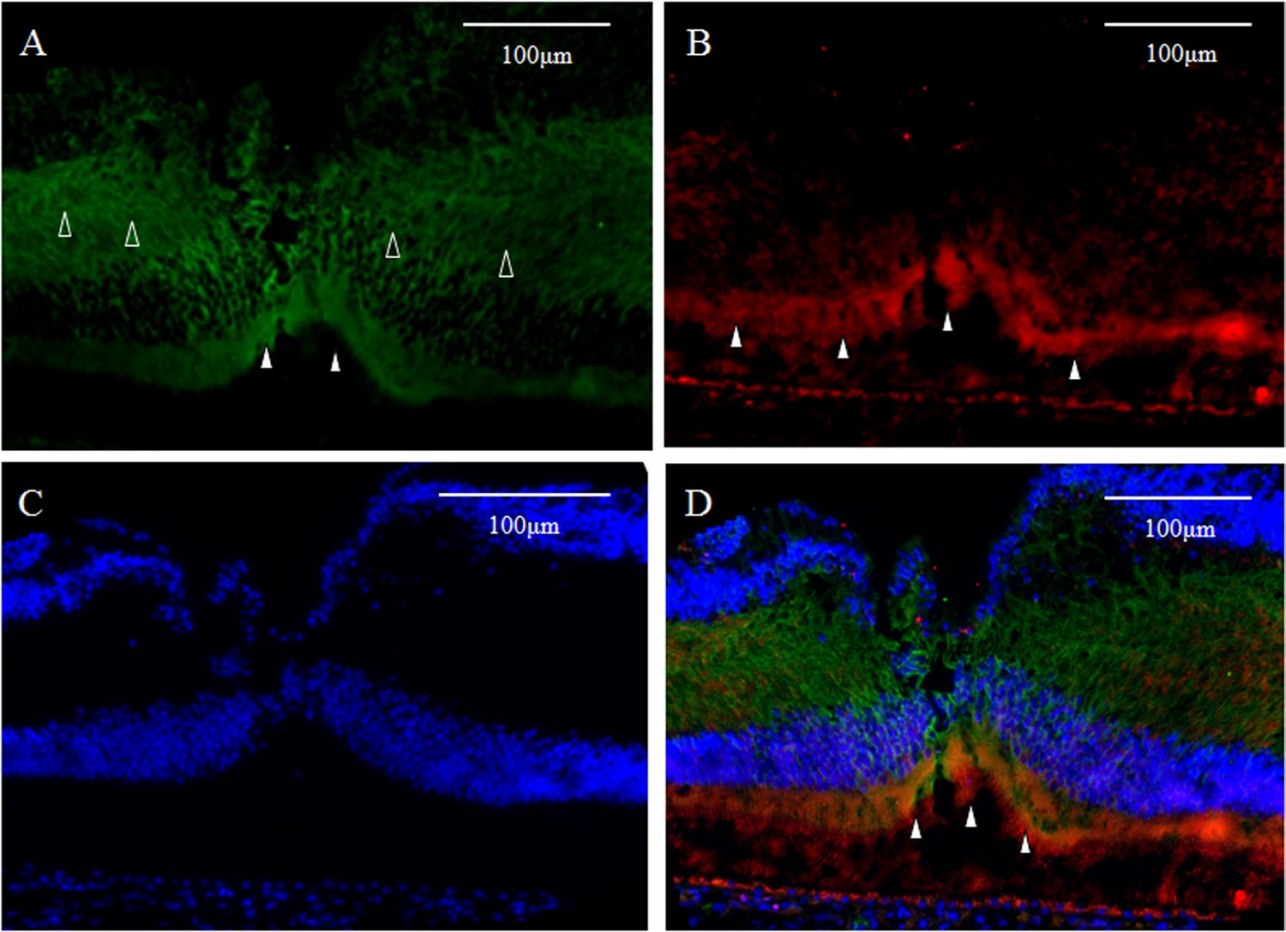
High-power-magnification optical microscopy images of a 4-year-old male monkey showing immunostaining of the foveola (vertical cross section) for nestin (green) and arrestin 4 (red). (**A**) Immunostaining for nestin (green) is mainly observed in the foveolar photoreceptor cell layer (white arrowheads) and the Henle layer (unfilled arrowheads). (**B**) Immunostaining for arrestin 4 (red) is mainly visible in the photoreceptor cell layer alone (white arrowheads). (**C**) DAPI staining (blue). (**D**) In the double-staining for nestin and arrestin 4 including the immunostaining for DAPI (blue), colocalization yellowish regions, is observed in the foveolar photoreceptor layer (white arrowheads).

Similarly, immunostaining for nestin (green) was observed mainly in the photoreceptor cell layer of the foveola (Fig. 10A, white arrowheads) and the Henle layer (Fig. 10A, unfilled arrowheads) of the fovea, and immunostaining for arrestin 4 (red), a marker of cone cells, was observed in the photoreceptor cell layer, both at and outside the foveola (Fig. 10B, white arrowheads), and showed definitive merging of nestin and arrestin 4 in the foveolar photoreceptor cell layer, which consists of cones alone (Fig. 10D, white arrowheads).

**Figure 11:**
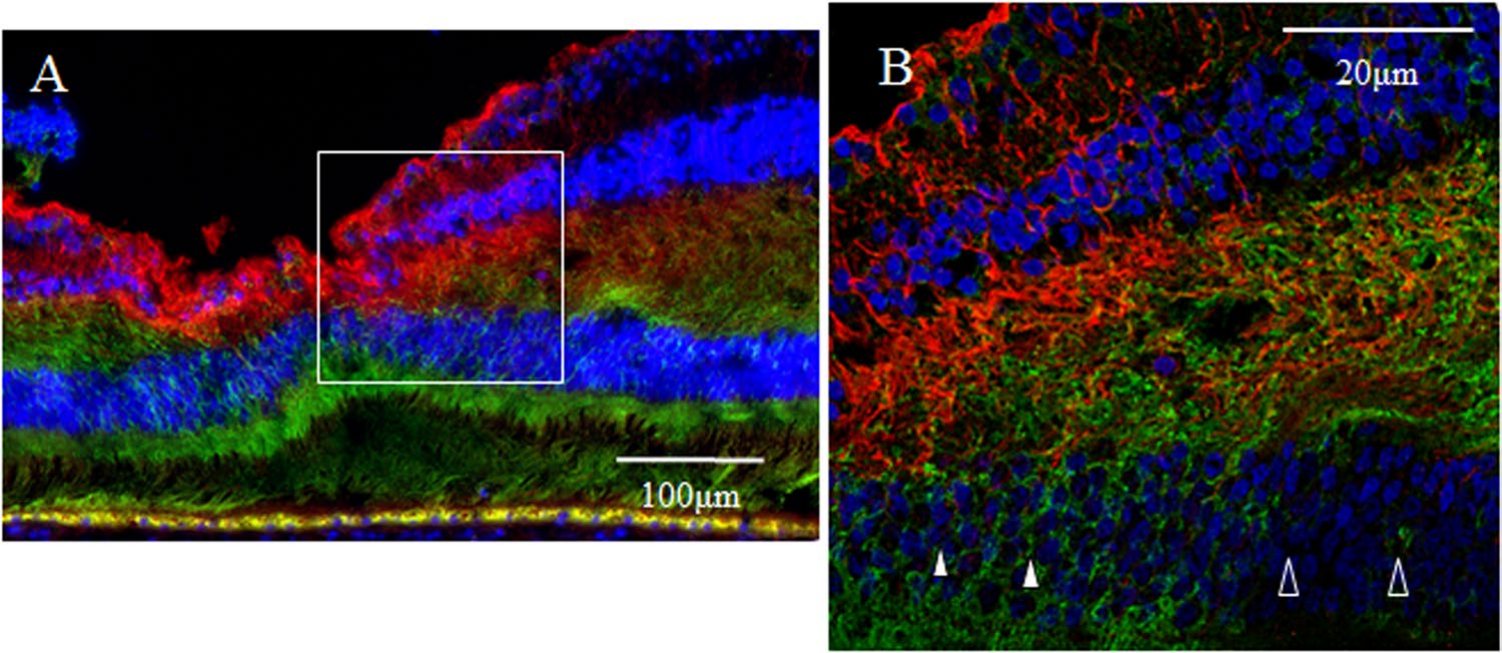
Confocal microscopy images of a 6-year-old female monkey showing immunostaining of the foveola (vertical cross section) for nestin (green) and GFAP (red). (**A**) Overview section of the foveola. The square indicates B. (**B**) The perinuclear area of the foveolar cone is richly stained for nestin (white arrowheads), yet the perinuclear area of the cone outside of the foveola shows weaker immunostaining for nestin (unfilled arrowheads).

In addition, the perinuclear area of the foveolar cone was richly stained for nestin (Fig. 11A,B, white arrowheads), yet the perinuclear area of the cone outside of the foveola showed weaker immunostaining for nestin (Fig. 11B, unfilled arrowheads).

### Nestin and Vimentin

**Figure 12:**
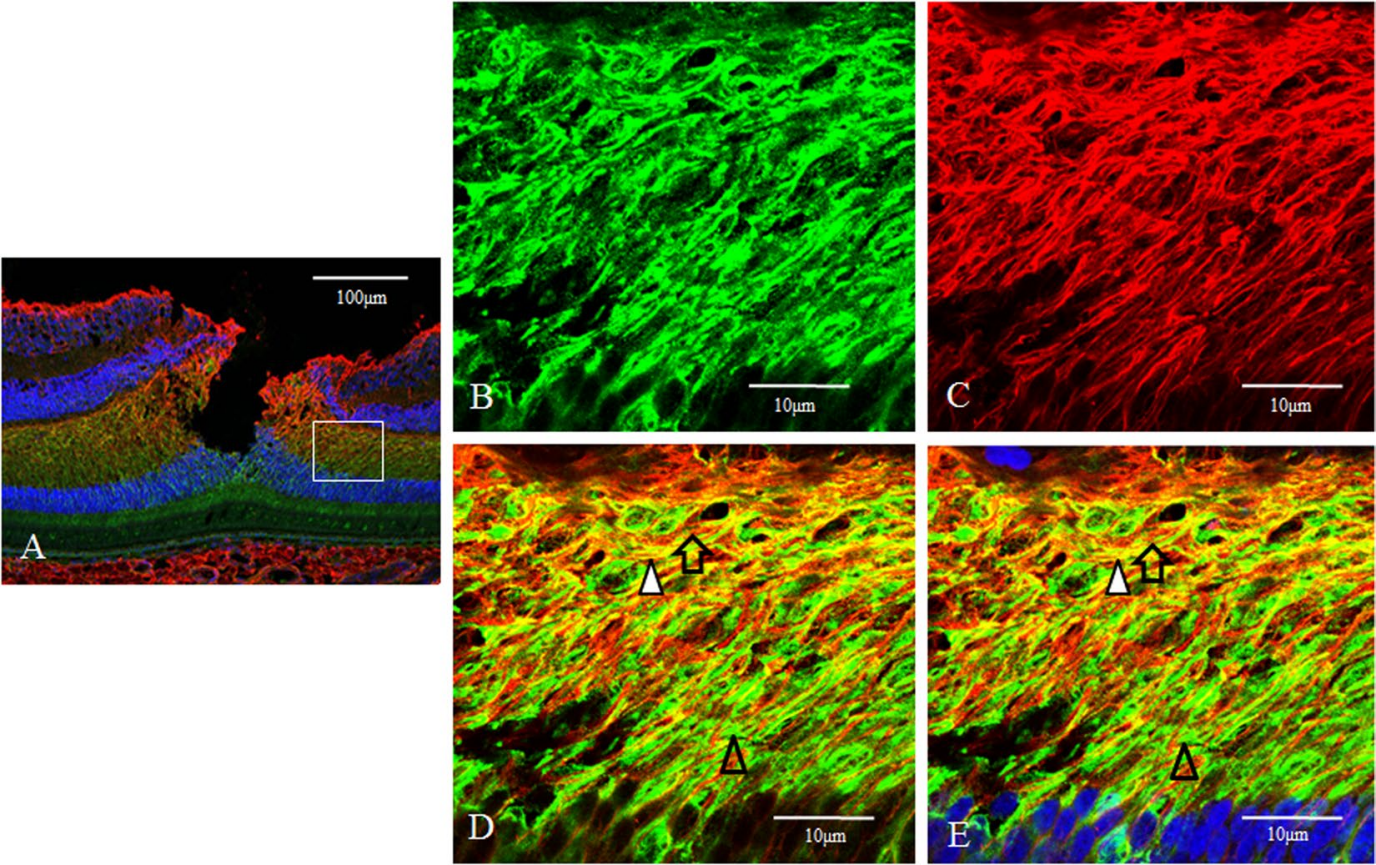
Medium-power-magnification optical microscopy and high-power-magnification confocal microscopy images of a 2-year-old male monkey showing double-immunostaining of the fovea (vertical section) for nestin and vimentin. (**A**) Medium-power-magnification optical microcopy images show the overview of the fovea. Yellowish regions, coexpression of nestin and vimentin, are observed mainly in the inner half of the Henle layer. (**B**) The expression of nestin (green) is uniformely observed in the Henle layer. (**C**) The expression of vimentin (red) is more prominent in the inner half of the Henle layer. (**D**,**E**) The double staining for nestin and vimentin reveals that yellowish fibrous processes, indicating the coexpresion of nestin and vimentin, are observed predominantly in the inner half of the Henle layer. Nnestin-single positive (green) and vimentin-single positive (red) processes are also observed. These three kinds of fibrous processes extend obliquely in the Henle layer.

Optical microscopy revealed that yellowish regions, indicating coexpression of nestin and vimentin, were observed only in the Henle layer, especially in the inner half (Fig. 12A). The expression of nestin (green) was uniformely observed in the Henle layer (Fig. 12B). The expression of vimentin (red) was more prominent in the inner half of the Henle layer (Fig. 12C). Confocal microscopy showed that three kinds of fibrous precesses, namely (1) nestin-single positive, (2) vimentin-single positive, (3) nestin-vimentin-double positive, extended obliquely in the Henle layer (Fig. 12D,E). In the vicinity of the inner nuclear layer, these three kinds of processes extended nearly horizontally. Some of these processes traversed the Henle layer.

### Nestin and Neurofilament

**Figure 13:**
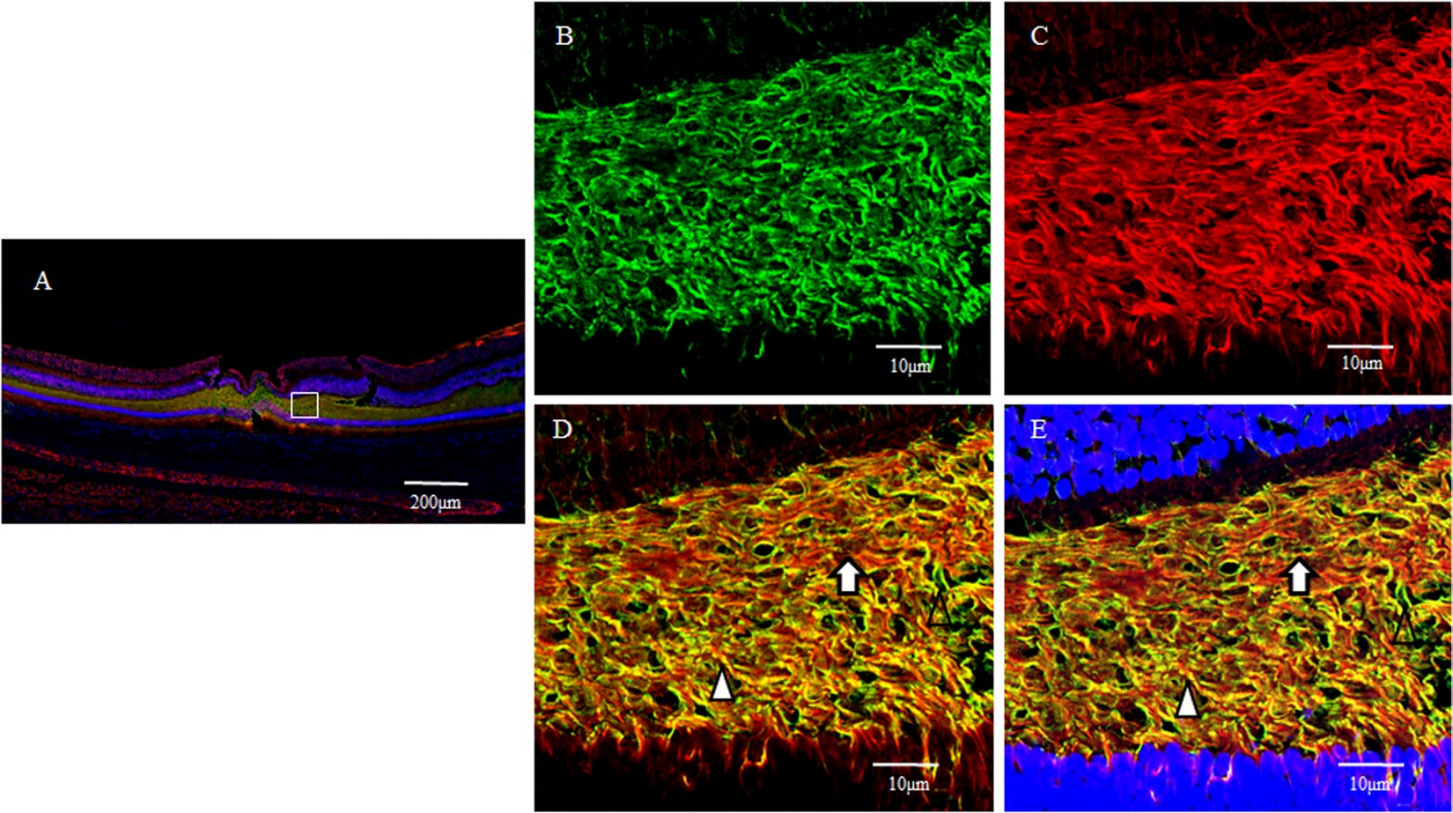
Low-power-magnification optical microscopy and high-power-magnification confocal microscopy images of a 2-year-old male monkey showing double-immunostaining of the fovea (vertical section) for nestin and neurofilament. (**A**) Low-power-magnification optical microscopy reveales that yellowish regions, indicating coexpression of nestin and neurofilament, are observed in the Henle layer. (**B**,**C**) Confocal microscopy showed that the expression of nestin (green) and neurofilament (red) are uniformely observed in the Henle layer. (**D**,**E**) In the double-staining for nestin and neurofilament, yellowish regions (white arrowheads), indicating coexpression of nestin and neurofilament, are more prominent in the outer half of the Henle layer. Reddish regions (white arrows), indicating the axon that do not express nestin, are more prominent in the inner half of the Henle layer. Nestin single positive regions, presumably Müller cell outer processe, are also partially observed in the Henle layer (unfilled arrowheads).

Optical microscopy revealed that yellowish regions, indicating coexpression of nestin and neurofilament, were observed in the Henle layer (Fig. 13A). Confocal microscopy showed that the expression of nestin (green) was observed uniformely in the Henle layer (Fig. 13B). The expression of neurofilament (red), a marker of axons, was also distributed uniformely in the Henle layer (Fig. 13C). In the double staining for nestin and neurofilament, yellowish regions, indicating coexpression of nestin and neurofilament, were more predominant in the outer half of the Henle layer (Fig. 13D,E, white arrowheads). On the other hand reddish regions, indicating the axons that did not express nestin, were more predominant in the inner half of the Henle layer (Fig. 13D,E, white arrows). Nestin-single positive greenish regions, presumably Muller cell outer processes, were also partially observed in the Henle layer (Fig. 13D,E, unfilled arrows).

### CD 117 and CD44

**Figure 14:**
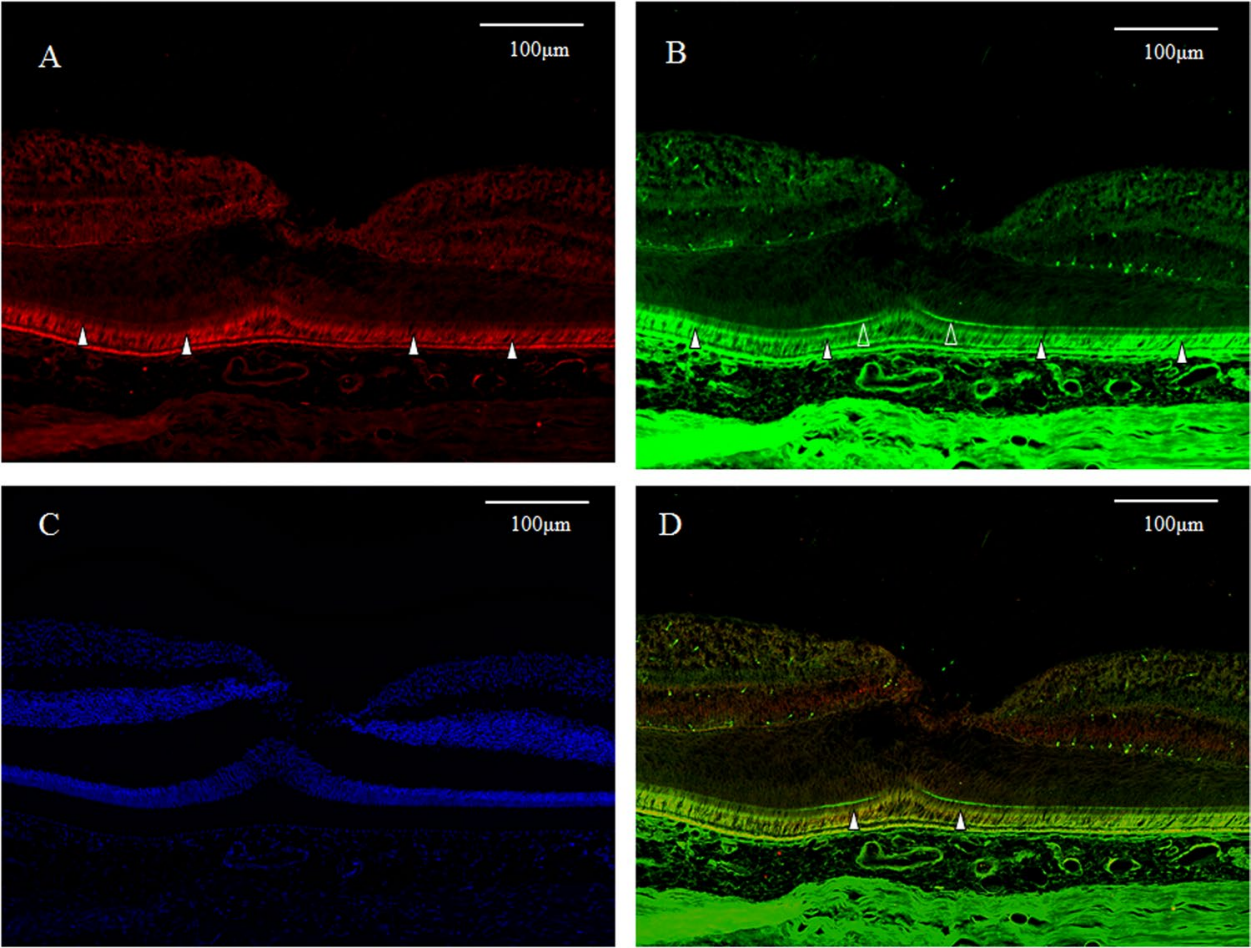
Medium-power-magnification optical microscopy images of a 4-year-old male monkey showing immunostaining of the fovea (vertical cross section) for CD 117 (red) and CD44 (green). (**A**) Immunostaining for CD117 is observed predominantly in interphotoreceptor matrix (white arrowheads). (**B**) CD44 is observed predominantly in Müller cell apical microvilli (unfilled arrowheads) in the foveal area and in the interphotoreceptor matrix (white arrowheads). (**C**) DAPI staining (blue). (**D**) In the double-staining for CD117 and CD44, colocalization, a yellowish region, is visible in the interphotoreceptor matrix of the fovea (white arrowheads).

Medium-power-magnification optical microscopy images revealed immunostaining for CD117 (red) was found predominantly in the interphotoreceptor matrix (Fig. 14A, white arrowheads). CD44 expression was found predominantly in the interphotoreceptor matrix (white arrowheads) and in the Müller cell apical microvilli (unfilled arrowheads) in the foveal retina, except the foveolar region (Fig. 14B). [DAPI staining (blue) (Fig. 14C)]. In the double-staining for CD117 and CD44, a yellow regions, indicating the merging of CD117 and CD44, was visible presumably in the foveal interphotoreceptor matrix (Fig. 14D, white arrowheads).

**Figure 15:**
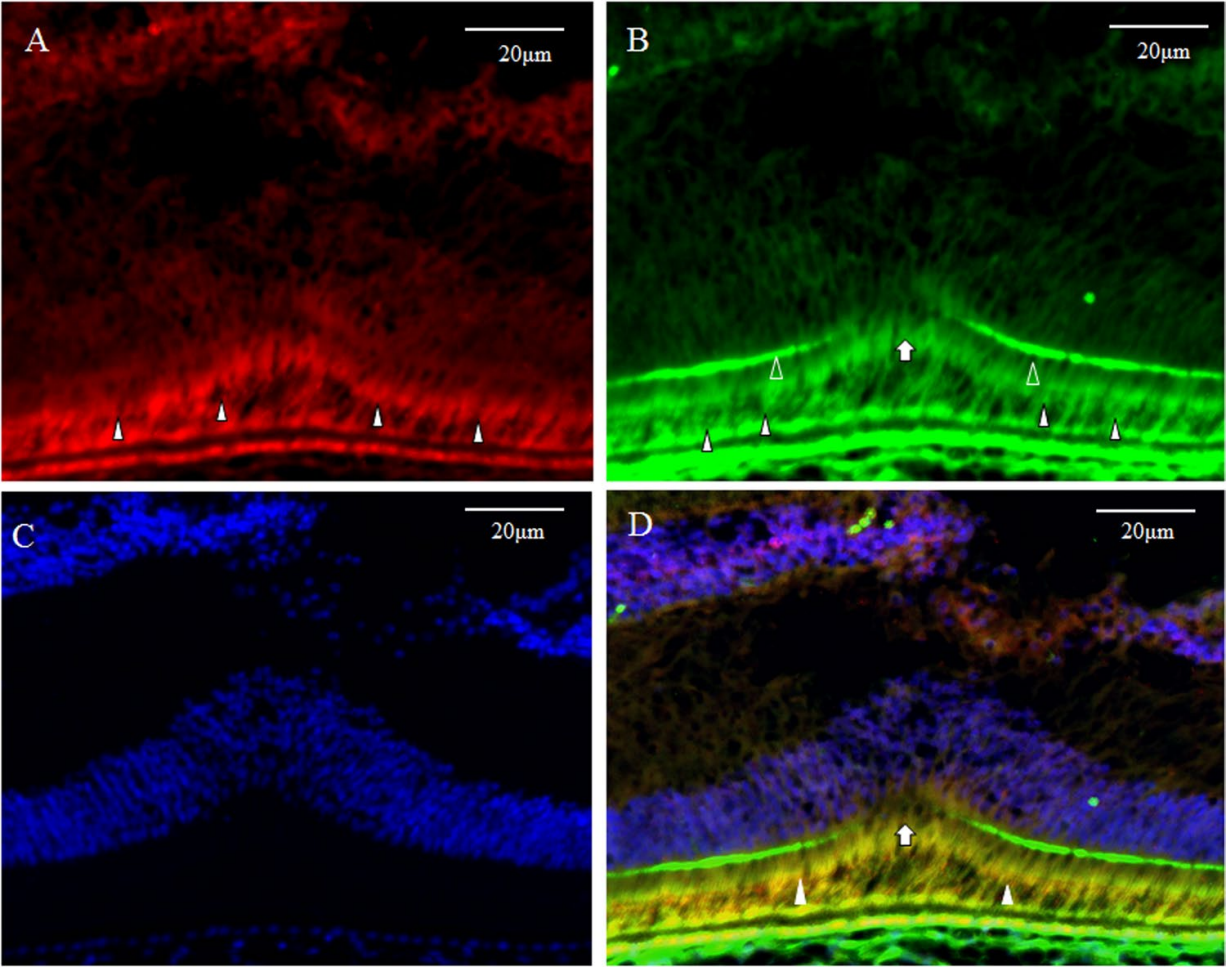
High-power-magnification optical microscopy images of a 4-year-old male monkey showing immunostaining of the fovea for CD 117 (red) and CD44 (green) in the foveola (vertical cross section). (**A**) Immunostaining for CD117 is found predominantly in the interphotoreceptor matrix (white arrowheads). (**B**) Immunostaining for CD44 is found predominantly in the Müller cell apical microvilli in the fovea (unfilled arrowheads) except the foveolar region (white arrow) and in the interphotoreceptor matrix (white arrowheads). (**C**) DAPI staining (blue). (**D**) In the double staining for CD117 and CD44, colocalization yellowish regions, is visible in the interphotoreceptor matrix of the fovea (white arrowheads). Discontinuity of Müller cell apical microvilli is observed in the foveolar region the foveolar region (white arrow).

High-power-magnification optical microscopy images showed more clearly the immunostaining for CD117 and CD44 in the interphotoreceptor matrix (Fig. 15A, white arrowheads). As described above CD44 expression which was found predominantly in the interphotoreceptor matrix (Fig. 15B, white arrowheads) and Müller cell apical microvilli in foveal retina except the foveolar region (Fig. 15B, unfilled arrowheads). [DAPI staining (blue) (Fig. 15C)]. In the double staining for CD117 and CD44, a yellowish region indicates the coexpression of CD117 and CD44 in the interphotoreceptor matrix of the fovea (Fig. 15D, unfilled arrowheads).

### Ki67 staining

**Figure 16:**
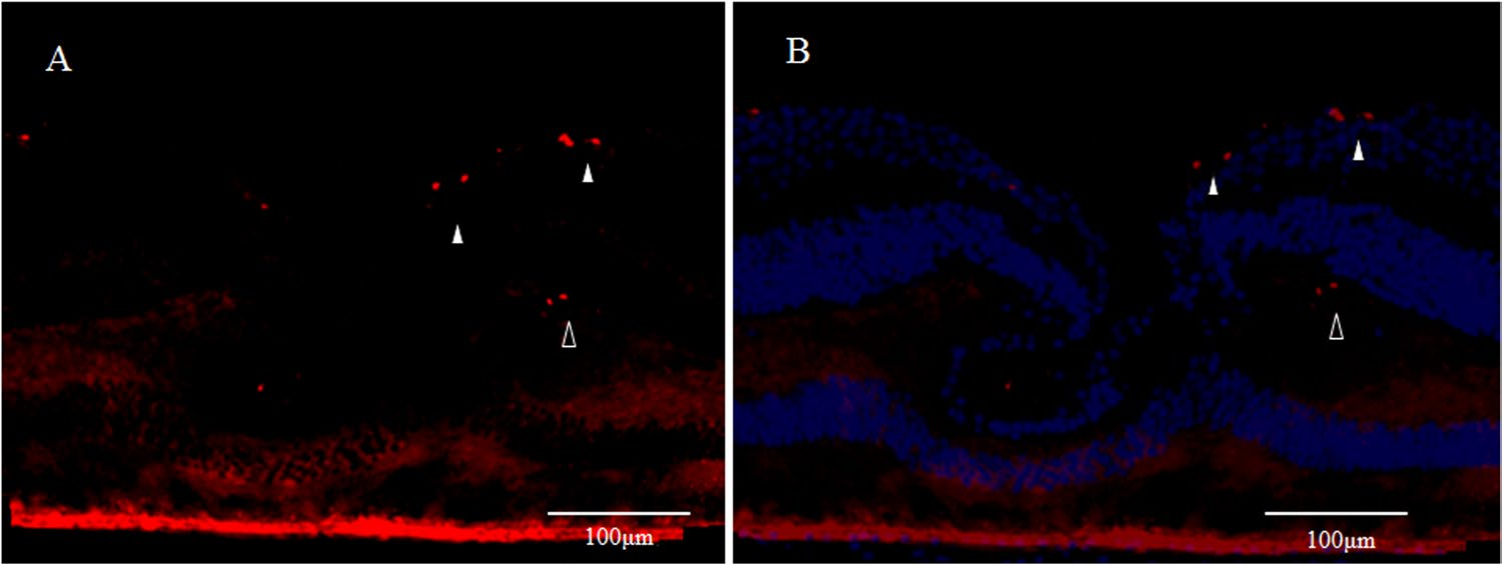
High-power-magnification optical microscopy images of a 4-year-old male monkey showing immunostaining of the fovea for Ki67. (**A**) Scattered Ki67-positive spots (red) are visible in the retinal innermost layer around the foveola (white arrowheads). Moreover, some parts of the region believed to be the outer plexiform near the inner nuclear layer around the foveola are also Ki67-positive (unfilled arrowheads). (**B**) Immunostaining for Ki67 includes the immunostaining for DAPI (blue).

Immunostaining for Ki67 (red) was positive in scattered spots in the ganglion cell layer at the foveal slope surface around the foveola (Fig. 16A,B, white arrowheads). In addition, positive staining for Ki67 was also observed in parts of the area believed to be the outer plexiform layer around the foveola (Fig. 16A,B, unfilled arrowheads).

### GFAP and cellular retinaldehyde-binding protein (CRALBP)

**Figure 17:**
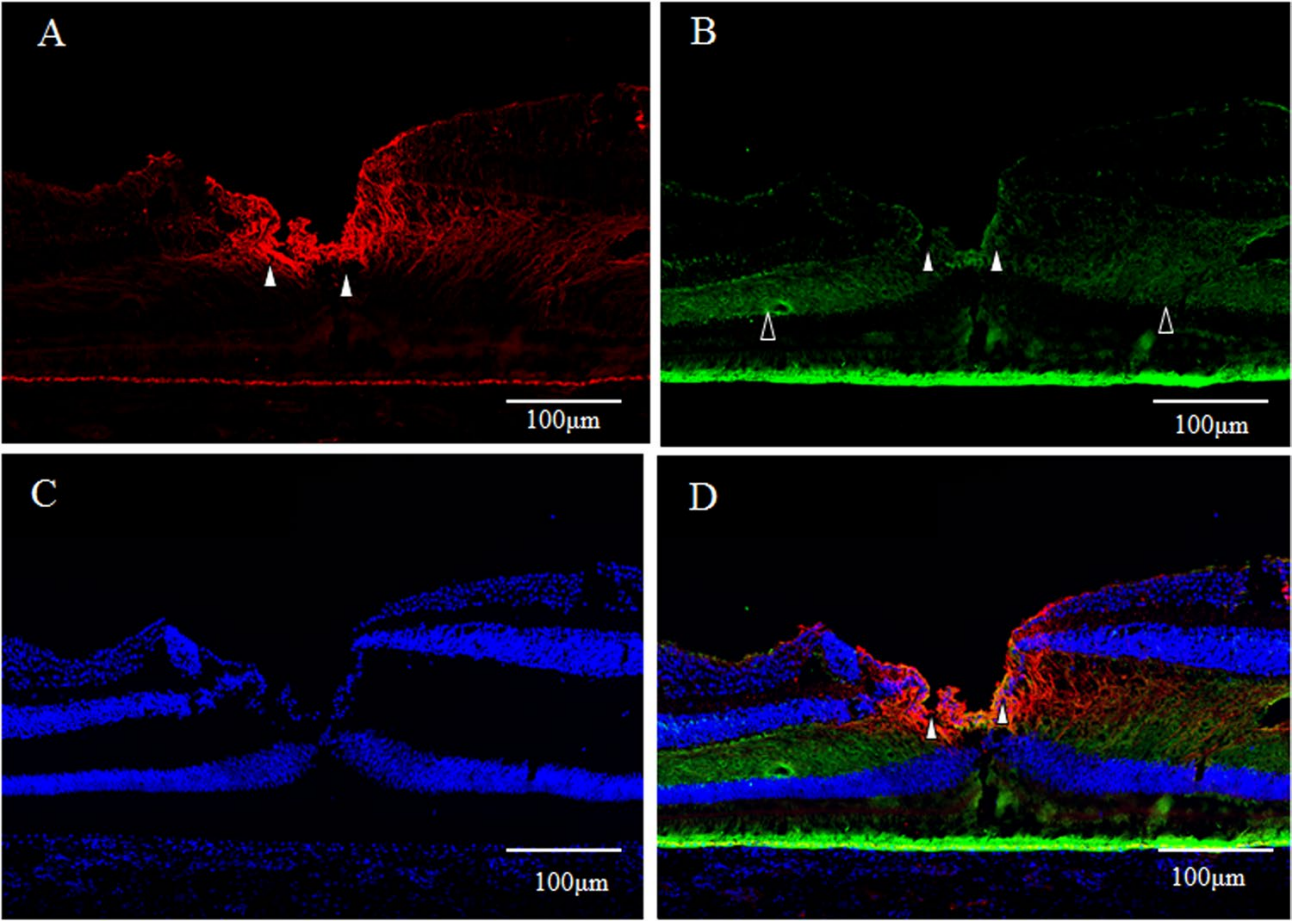
High-power-magnification optical microscopy images of a 6-year-old female monkey showing immunostaining of the fovea for GFAP and cellular retinaldehyde-binding protein (CRALBP). (**A**) GFAP (red) expression is visible as intense staining in the inner layer of the foveal Müller cell cone (white arrowheads), the same as is shown in Fig. 1. (**B**) Immunostaining for CRALBP (green) is slightly observed in the innermost layer of the Müller cell cone (white arrowheads), yet it was negative in most parts of the GFAP-positive area. Moreover, CRALBP staining is visible in the outer plexiform layer around the foveola (unfilled arrowheads). (**C**) DAPI staining (blue). (**D**) In the double-staining for GFAP and CRALBP, almost no colocalization is visible in the inner layer of Müller cell cone of the foveola (white arrowheads).

Intense immunostaining for GFAP expression (red) was similarly observed in the inner 7layer of the Müller cell cone in the foveola (Fig. 17A, white arrowheads). Slight immunostaining for CRALBP (green) was observed in parts of the innermost layer of the Müller cell cone (Fig. 16B, white arrowheads), yet it was negative in most parts of the GFAP-positive area. Moreover, staining for CRALBP was observed centered in the outer plexiform layer around the foveola (Fig. 17B, unfilled arrowheads). [DAPI staining (blue) (Fig. 17C)]. Double-staining revealed almost no merging of GFAP and CRALBP in parts of the inner layer of the Müller cell cone in the foveola (Fig. 17D, white arrowheads).

## Discussion

The fovea, including the foveola, plays an important role in the perception of both form and color, among the other functions of the photoreceptor cells, and is involved in various diseases such as macular hole, epiretinal membrane, and macular edema. In recent years, the evolution of optical coherence tomography (OCT) has provided in-depth of morphological understanding on the macular diseases, and has prompted efforts to elucidate the causes of various macular diseases based on OCT findings. As reported by Gass in 1999, the theoretical basis for such efforts is the hypothesis that the Müller cell cone present in the foveola is involved in the pathogenesis of various macular diseases^21^.

Gass’s hypothesis was based on the optical and electron microscopy examinations of the foveola of a 45-year-old woman performed in 1969 by Yamada, who reportedly observed the presence in the foveola of an inverted cone-shaped aggregate of Müller cells that spanned to the area around the foveola on the side of the internal limiting membrane and to the external limiting membrane of the foveola^22^. Prior to the publication of Yamada’s study, Müller cells were thought to either not exist in the foveola^23^ or, in fact, practically be absent or discontinuous in the external limiting membrane of the foveola formed by the adjunct junctions between Müller cells and photoreceptor cells^24^. Previous histological studies revealed that the light cytoplasm of Müller cells was seen in the center of the foveola^25^. Syrbe *et al*. investigated the ultrastructure of the Müller cells in the foveola of both human and macaque retinas, and their findings revealed that there are various conspicuous features of foveolar Müller cells^26^. In this present immunohistological study, staining with a single cell marker failed to identify the aggregate of cells in the foveola with the internal limiting membrane as its base and spanning to the outer limiting membrane, as previously described by Gass and Yamada. However, intense immunostaining for GFAP was observed in the area corresponding to the inner-half layer of the Müller cell cone, yet weak immunostaining for GFAP was observed in the outer-half of Müller cell cone located in the photoreceptor layer. It should be noted that Powner *et al*. reported similar results in a study of the human foveola using immunostaining for GFAP in the inner layer of the Müller cell cone^27^.

In this present study, we also performed immunostaining for vimentin, which is a marker of Müller cells and another marker of neural stem cells, and found that weak double-staining for GFAP and vimentin was observed in the surface of the foveal slope. Powner *et al*. and Bringmann *et al*. reportedly observed immunoreactivity with GFAP and vimentin in the fovea^25,27^. The results of those studies showed that the expression for vimentin was observed in the area around the foveola and the expression for GFAP was observed in the inner layer of foveola. Our results revealed the almost identical pattern to that in those reports, however we observed the colocalization of GFAP and vimentin at the foveal slope. De Guevara *et al*. reported that both vimentin and GFAP were early expressed in the developing retina and, particularly in the Müller cells, a coexpression of vimentin and GFAP was observed from embryonic to adult stages^28^. Bringmann *et al*. also reported that z-shaped Müller cells around the foveola were vimentin and GFAP double positive^25^. Those results indicate that the area around the foveola might be composed of undifferentiated Müller cells.

In primate retinas, astrocytes display immunostaining for GFAP, yet Müller cells do not, except those that are undifferentiated Müller cell during the fetal stage and reactive Müller cells that appear after tissue injury^29^. On the other hand, astrocytes in the optic disc and around the peripapillary blood vessels in the nerve fiber layer are known to display strong immunostaining for GFAP^30^. Our findings indicated that the inner layer of the foveola (*i.e*., the inner-half layer of the Müller cell cone) showed a GFAP staining pattern that well-resembled that displayed by astrocytes in the peripapillary area.

The aggregate of GFAP-positive cells spanned from the inner layer of the foveola, in a horizontal direction through the retinal surface layer, to the area around the foveola. It further elongated along the deep retinal capillary plexus at the boundary between the inner nuclear layer and the outer plexiform layer to the foveal periphery. This horizontal elongation is a characteristic that closely resembles that of astrocytes in the optic disc periphery, but different from that of Müller cells, which span radially to cover the entire retinal layer. Moreover, on a horizontal cross-section of the foveal retina, GFAP-positive elongated projections that spanned toward, and clung to the blood vessels, which is also a morphological characteristic of astrocytes, were observed. Staining with cell markers other than GFAP showed that almost no immunostaining for CRALBP, a reported marker for differentiated Müller cells^31^, was present in the inner layer of the foveola. The findings in previous reports have indicated that CRALBP and GFAP are never expressed concurrently^32,33^. Thus, we believe that the GFAP-positive area in the inner layer of the Müller cell cone is comprised of astrocytes rather than Müller cells.

The distribution of xanthophylls also supports the argument that the GFAP-intensely positive cells are comprised of astrocytes. Reportedly, xanthophylls are macular pigments that are distributed consistently to the foveal pit area, *i.e*., the inner-half layer of the Müller cell cone^34^. Moreover, xanthophylls such as lutein and zeaxanthin, after binding with high-density lipoprotein (HDL), are taken up intracellularly through scavenger receptors such as scavenger receptor class B type I (SR-BI)^35^. Astrocytes reportedly express SR-BI^36^, however, and to the best of our knowledge, there has been no report that Müller cells express an SR-BI that mediates HDL uptake. Furthermore, the tubulin, which is known to bind with xanthophylls^37,38^, is widely distributed intracellularly in astrocytes^39^. These facts are also consistent with the findings that macular pigments are present uniformly in the inner-half layer of the Müller cell cone.

In addition, confocal microscopy revealed the presence of GFAP-positive cells, although slightly weakly stained, spanning radially from the inner layer of the above-described Müller cell cone up to the photoreceptor cell layer. Anatomically, astrocytes in the brain include large protoplasmic astrocytes that are present in the gray matter, fibrous astrocytes that are present in the white matter, with elongated projections reaching into small spaces among surrounding nerve cells, and radial glial cells that are present during the developmental stage and that are responsible for guiding neuron migration^40^. Radial glia cells reside in the place where neurogenesis occurs in adult CNS. The first two are embedded deeply into the gray matter and the white matter, respectively, whereas radial glial cells are characterized by their projections spanning to the cerebral ventricle side and the cerebral pial side^41^. It should be noted that there is still room for further study as to whether or not the GFAP-weakly-positive region, observed running vertically in the foveola under confocal microscopy in this study, represents radial glial-like cells. Nevertheless, if adult neurogenesis occurs in the foveola, astrocytes and radial glial cells may both be present in the foveola as same as in the CNS.

The findings in previous reports have indicated that astrocytes are present where neurogenesis occurs in the CNS, and that they assist neurogenesis at that site^42,43^, thus suggesting that the GFAP-strongly-positive astrocytes on the inside of the foveola may assist neurogenesis in that vicinity, just as in the area of the brain where neurogenesis occurs. Dutheil *et al*. described that neurogenesis and astrogliogenesis contribution to recovery of vestibular functions following unilateral vestibular neurectomy in an adult cat model^44^. In addition, Widestrand *et al*. reported increased neurogenesis and astrogliogenesis from neural progenitor cells grafted in the hippocampus of mice^45^.

In this present study, a layer of Tuj-1-positive cells, which also partially stained for GFAP, was observed spanning from the foveolar surface layer to the area around the foveola, and continued to the multilayered ganglion cell layer. Tuj-1 is reportedly a marker of ganglion cells and other neurons^46^. However, anatomically, ganglion cells are said to be absent in the foveola^47^. Nevertheless, given that nuclei were known to be present in the foveolar surface layer^48^, such cells were probably heterotopic ganglion cells. Böhm *et al*. reported that the foveola in humans and in rhesus monkeys displayed immunostaining for Tuj-1^49^. Tuj-1-positive cells in this area are probably ganglion cells, or their precursor. In this study, the area of the foveal slope showed scattered Tuj-1-positive cells that displayed immunostaining for Ki67, a marker of cell division, indicating that the division of Tuj-1-positive precursors of ganglion cells in the foveola may lead to neurogenesis and contribute to the homeostatic regeneration of the fovea. Alternatively, astrocytes in the center of the foveola may be in a given astrogliogenesis, and it has also been reported that astrocytes during the embryonic period express both Tuj-1 and GFAP^50^.

Tuj-1 is a marker of differentiated neurons or neuroblasts, thus indicating that these cells do not have the properties of stem cells that can self-renew^51^ and suggesting that the Tuj-1-positive cells differentiated from precursor cells. We believe that the nestin-positive foveolar cone is a corresponding candidate. In a previous study, we performed immunostaining of cynomolgus monkey eyes for nestin, a neural-stem-cell marker and a cytoskeletal intermediate filament, in sections of the fovea, mid-periphery, equator, and extreme periphery^19^. Our comparison of the nestin-positive cell density in each region clearly showed a higher density in the fovea, but compared with the other regions. In addition, we performed real-time PCR to analyze differences in the expressions of neural-stem-cell-related genes in different regions of the monkey retina (*i.e*., macula, midperiphery, and extreme periphery), and our findings confirmed high levels of nestin expression in the macula^20^.

The findings in this present study revealed positive staining in the foveolar photoreceptor layer for nestin and arrestin 4, a marker of cones, yet weak staining outside of the foveola. Generally, the outer segment of the foveolar cone is long^52^, and appears as a raised ellipsoid zone even on OCT imaging^53^. The cones in this region alone are different from those in the vicinity and presumably show the properties of undifferentiated cells, which may have resulted in the intense immunostaining for nestin. Moreover, confocal microscopy confirmed the presence of nestin at the nuclear periphery of the foveolar cone, which is a characteristic of mitotically-active cells^54^. Nestin is a marker of neural stem/progenitor cells, and its expression has been detected in regions where neurogenesis occurs, including the subventricular zone and the hippocampal subgranular zone in the brain^55^, thus suggesting that the foveolar cone may have the characteristics of neural stem cells. In the fovea, which is subjected to intense photo-stress, the number of cones is maintained throughout a person’s life^56^, whereas the number of rods decrease with aging. Several studies have also reported that rods are frangible by oxidative stresss and photo damage, while cones are more resistant to them^57–59^. However this theory is based on the cell density throughout the life of rod and cone. Some studies have reported that cell death of cones was observed by oxidative stresss and photic injury as well as rods^60–63^. These findings suggest that the number of the foveal cones may be maintained by homeostatic regeneration.

Optical microscopy revealed that besides in the foveolar cones, nestin was uniformly observed in the Henle layer as described before. Vimentin, a marker of Müller cells, was observed around the foveola, including in the Henle layer especially in the inner half. Colocalization of nestin and vimentin was observed only in the Henle layer, predominantly in the inner half. Confocal microscopy showed nestin-single positive, vimentin-single positive and nestin/vimentin-double positive fibrous processes extended obliquely in the Henle layer, and some of these three types of processes traversed the entire layer. The Henle layer comprises axons of the foveal cones and Müller cell outer processes. Nestin-single positive fibrous processes might be axons of the foveal cones, because they didn’t express vimentin, a marker of Müller cells. It was reported that nestin was expressed in the growing axon^64–66^ and in the growth cone. In the olfactory system, neurogenesis and axonal regeneration occurred continuously throughout life^67,68^. If neurogenesis continues in the adult foveal region, axonal regeneration might also occur in the foveal cones, resulting in the expression of nestin in their axons. Nestin-single positive fibrous processes were predominant in the outer half of the Henle layer, presumably because growing axons of the foveal cones accumulated there. It was reported that nestin expressed in Müller cells of the fetal retina, however, nestin-expressing Müller cells were almost completely diminished as maturation proceeded, except in the case of tissue injury^69,70^. If immature Müller cells reside around the foveola, as previously described, nestin is presumably observed in their outer processes. The existence of vimentin-single positive and nestin/vimentin-double-positive processes in the Henle layer supposedly indicated that immature Müller cells resided in the foveal region and intermingled with mature Müller cells. Nestin/vimentin-double positive Müller cell outer processes were more abundant in the inner half of the Henle layer, presumably because the growing outer processes of immature Müller cells might accumulate there.

Moreover, confocal microscopy showed that coexpression of nestin and neurofilament were observed in the fibrous processes that extended in the Henle layer. Neurofilaments are intermediate filaments and are present in neurons, particularly abundant in axons, therefore, coexpression of nestin and neurofilament indicates that nestin exists in the axons of the foveal cones^71^. Coexpression of nestin and neurofilament were more prominently observed in the outer half of the Henle layer. As mentioned before, nestin expresses in the growing axon and in the growth cone. Therefore, axons in this area were conceivable as growing state. On the other hand neurofilament-single positive fibrous processes were more prominent in the inner half of the Henle layer, which indicated axons in this area were mature and formed synaptic connections. Nestin-single positive fibrous processes were also observed partially in the Henle layer. They were presumably Müller cell outer processes, because they did not express neurofilament, a marker of axons. Thus, the results of double staining for nestin and vimentin and those for nestin and neurofilament were complementary each other.

We also performed immunostaining for CD44 and CD117 in the macular retina. Too *et al*. reported the similar studies and they described that CD117 expression was observed in Müller cell cytoplasm spanning from inner to outer limiting membrane in both peripheral retina and macular retina. By contrast, CD44 expression was found predominantly in the Müller cell apical microvilli of peripheral retina, and no expression was found in macular retina^69^. However, our findings clearly showed an expression of CD44 in the Müller cell apical microvilli in the fovea except the foveolar region. Felemban *et al*. reported that CD44 play an important role in the development of photoreceptors and interphotoreceptor matrix^70^. These findings were similar to CD44 expression of the peripheral retina reported by Too *et al*., thus suggesting that consequently these foveolar cones might have the characteristics of neural stem cells to participate with regeneration.

It has been reported that two types of stem cells are present in small intestinal crypts, *i.e*., crypt-base-columnar (CBC) cells, which are active stem cells that divide vigorously, and +4 label-retaining cells (+4LRCs), which are slowly cycling quiescent stem cells^72^. Bmi-1, a marker for +4LRCs, is reportedly expressed in cones^73^. The center of the photoreceptor cells in the foveola are comprised of only long-wavelength and middle-wavelength cones (*i.e*., ‘L/M’ cones)^74^. Gene analyses have indicated that the L/M-cone precursor cells are the origin of retinoblastoma^75^. Genetic mutations in tissue stem cells or progenitor cells are believed to cause cancer^76^. Thus, the L/M cones within the foveolar photoreceptor cells, in particular, may have the properties of tissue stem cells. Thyroid hormone receptor β2 is reportedly involved in the expression of L/M-cone opsin^77^, and the thyroid hormone reportedly has the ability to reprogram the differentiated cells^78^, thus suggesting that L/M cones may dedifferentiate from cone precursors in the terminal differentiation process and thereby display the properties of stem cells.

Concavities, such as the small intestinal crypts and hair follicles^79^, as well as hypoxic regions such as the endosteal niche of bone-marrow stem cells^80^, are known to be sites where stem cells reside. As stated above, small intestinal crypts include two types of stem cells. One with fast cell cycle and one with slow cell cycle. In addition, hair follicles and bone marrow are known to also contain such two types of stem cells, thus allowing tissue homeostasis to be maintained. The bone marrow has a vascular niche providing a high-oxygen-partial-pressure environment near the sinusoid and an endosteal niche providing a low-oxygen environment in the periosteal surface layer^80^. The foveolar L/M cones, which are located most distant from the retinal vessels, express Bmi-1, a marker of quiescent stem cells in the intestinal crypts, thus suggesting that they are likely involved in tissue regeneration with slow cell cycle. Our study showed the Müller cells around the foveola had features of undifferentiated cell type, namely coexpression of GFAP and vimentin, and CD44 expression in the apical microvilli. Therefore we speculated that the Müller cells around the foveola could be stem cells, with fast cell cycle and contribute to epimorphic regeneration, such as closure of macular hole.

Previous reports have indicated that Müller cells showed the characteristics of retinal stem cells^81,82^, and that they were also involved in the regeneration of rods and cones in humans^83^. In humans, the foveal avascular zone measures 0.5 mm in diameter, versus that of 0.35 mm for the foveola^84^, and the oxygen level in the Müller cells in this ring-shaped region between the two circles is assumed to be lower than in other regions. It has been reported that hypoxia stimulates stem cells in tissues that express HIF-1α and increases stemness, thus leading to multipotency and self-renewal^85^. The Müller cells in this region may have coexpressed GFAP and vimentin, and expressed CD44 in the apical microvilli in our study. Glycinergic amacrine cells, which express Lgr5, a fast cycling stem cell marker of intestinal crypts, are another retinal stem cell candidate around the foveola^86^. These hypoxic Müller cells or glycinergic amacrine cells may contribute to homeostatic regeneration to maintain cell density constant and epimorphic regeneration of the foveola to close the macular hole.

In primates, the fovea is occupied by astrocytes at birth, however, they reportedly disappear shortly after birth^87^. It has been considered that astrocytes in the retina migrate from the optic disc, and that they are absent in adult fovea^88,89^. However, astrogliogenesis also occurs in the hippocampus and the subventricular zone, where the neurogenesis still occurs in adulthood^90–92^. In this present study, the GFAP-positive area in the foveola is increased with age (2-years old; Fig. 7, 4-years old; Figs 8 and 9, 6–years old; Figs 1, 3, 4 and 17), thus indicating that the astrogliogenesis might occur in the foveola of primates after birth.

We conducted this present study with the hypothesis that undifferentiated retinal stem cell-like cells are present in the foveola and its vicinity, which is concave and hypoxic, and anatomically characteristic of stem cell niches. The fovea, including the foveola, is a region where the most diverse remodeling processes in the retina occur due to disease, thus leading to macular hole, epiretinal membrane, macular edema, and other diseases. Nonetheless, immunohistological studies of the fovea, except those on photoreceptor cells, are rare due to technical difficulties. In fact, and to the best of our knowledge, the investigations performed in this present study are the first to focus on the immunostaining of the foveola with a marker of neural stem cells. We theorize that the findings in this study will provide valuable information that may help to elucidate the unique characteristics of the foveola, as well as the pathophysiology of various macular diseases.

## Materials and Methods

### Preparation of the monkey retinal tissue sections

The eyes of cynomolgus monkeys (2 males and 2 females; age range: 2–6 years) were purchased from Shiga Research Center, Nissei BILIS (formerly titled, Environmental Biological Life Science Research Center Inc.), Shiga, Japan. The body weight of these monkeys ranged from 2.8–3.2 kg. All monkeys were housed in cages (1.0 × 1.2 × 1.2 meters in size) at a room temperature of 23.0–26.8 °C and 40–70% humidity, with a 12-hour light/dark cycle. All animals had free access to normal diet food obtained from Oriental Yeast Co. (Osaka, Japan) and free access to water. Anesthesia was used at 10 mg/kg of ketamine, and euthanasia was done by intravenous injection of a lethal dose of pentobarbital. All procedures involving animals were conducted in accordance with the Guidelines for the Care and Use of Laboratory Animals at Nissei BILIS (No.9751). The monkey eyeballs were fixed in 4% paraformaldehyde phosphate buffer solution for 24 hours. In each fixed eyeball, the cornea and lens were removed to prepare eyecups, from which the vitreous body attached to the retina was removed via the use of tweezers. Next, the eyecups were immersed in 30% sucrose for 48 hours, and then embedded in optimal cutting temperature compound (Tissue-Tek^®^ O.C.T.; Sakura^®^ Finetek Japan Co., Ltd., Tokyo, Japan). The retinal tissues were then cut vertically and horizontally into thin frozen sections that included the foveola, optic disc, and extreme periphery of the retina with cryostat. This study was approved by the Osaka Medical College Committee on the Use and Care of Animals (Approval Number: 26109).

### Immunostaining of tissue sections

The above-described thin foveolar sections were incubated with the primary antibodies of glial fibrillary acidic protein (GFAP) (1:500; Thermo Fisher Scientific, Fremont, CA or BioLegend^®^, Inc., San Diego, CA), nestin (1:500; Merck Millipore, Billerica, MA), vimentin (1:500; Abcam, Cambridge, UK), rabbit polyclonal anti-neurofilament M antibody (1:200; Merck Millipore, Darmstadt, Germany), neuron-specific class III β-tubulin (Tuj-1) (1:500; Covance Inc., Princeton, NJ), CD117(1:500; Abcam, Cambridge, UK), CD44 (1:500; Abcam, Cambridge, UK), Ki67 (1:500; Abcam Plc, Cambridge, UK), cellular retinaldehyde-binding protein (CRALBP) (1:500; Abcam), and arrestin 4 (1:500; Bioworld Technology, Inc., St. Louis Park, MN) for 48 hours at 4 °C. In addition, chemical reaction for DAPI (Dojindo Laboratories, Kumamoto, Japan) and double-immunostaining for GFAP and nestin, GFAP and Tuj-1, nestin and arrestin 4, nestin and vimentin, nestin and neurofilament, CD117 and CD44, and GFAP and CRALBP, respectively, was performed. The primary antibodies in this study were used in accordance with the methods described in the previous reports by us and others. These antibodies were evaluated by the experiments using knock-out animals.

These sections were incubated for 2 hours at room temperature in Alexa 594 or Alexa 488-conjugated to the appropriate secondary antibodies (1:1000; Invitrogen Corporation, Carlsbad, CA). Each section was examined via fluorescence microscopy (BZ-X700; Keyence Corporation, Osaka, Japan), and the sections that included the foveola were photographed. Moreover, sections double-stained for GFAP and nestin, and GFAP and Tuj-1, respectively, were examined via confocal microscopy (TCS SP8; Leica Microsystems GmbH, Wetzlar, Germany) and photographed with a high-sensitivity charge-coupled device (CCD) camera (DP30BW; Olympus Corporation, Tokyo, Japan) controlled by MetaMorph^®^ Microscopy Automation and Imaging Analysis Software (Universal Imaging Corporation, West Chester, PA). Thin sections of the optic disc and the extreme periphery of the retina were immunostained for GFAP and nestin, simultaneously in combination with DAPI.
